# RecruitPlotEasy: An Advanced Read Recruitment Plot Tool for Assessing Metagenomic Population Abundance and Genetic Diversity

**DOI:** 10.3389/fbinf.2021.826701

**Published:** 2022-01-27

**Authors:** Kenji Gerhardt, Carlos A. Ruiz-Perez, Luis M. Rodriguez-R, Roth E. Conrad, Konstantinos T. Konstantinidis

**Affiliations:** ^1^ School of Biological Sciences, Atlanta, GA, United States; ^2^ Center for Bioinformatics and Computational Genomics, Atlanta, GA, United States; ^3^ Department of Microbiology and Digital Science Center (DiSC), University of Innsbruck, Innsbruck, Austria; ^4^ Ocean Science & Engineering, Atlanta, GA, United States; ^5^ School of Civil and Environmental Engineering, Georgia Institute of Technology, Atlanta, GA, United States

**Keywords:** bioinformatics, software, metagenomics, MAG, population diversity

## Abstract

Mapping of short metagenomic (or metatranscriptomic) read data to reference isolate or single-cell genomes or metagenome-assembled genomes (MAGs) to assess microbial population relative abundance and/or structure represents an essential task of many studies across environmental and clinical settings. The filtering for the quality of the read match and assessment of read mapping results are frequently performed without visual aids or with the assistance of visualizations produced through ad-hoc, in-house approaches. Here, we introduce RecruitPlotEasy, a fully automated, user-friendly pipeline for these purposes that integrates statistical approaches to quantify intra-population sequence and gene-content diversity and identify co-occurring relative populations in the sample. Hence, RecruitPlotEasy should also greatly facilitate population genetics studies.

RecruitPlotEasy is implemented in Python and R languages and is freely available open source software under the Artistic License 2.0 from https://github.com/KGerhardt/RecruitPlotEasy.

## Introduction

**FIGURE 1:**
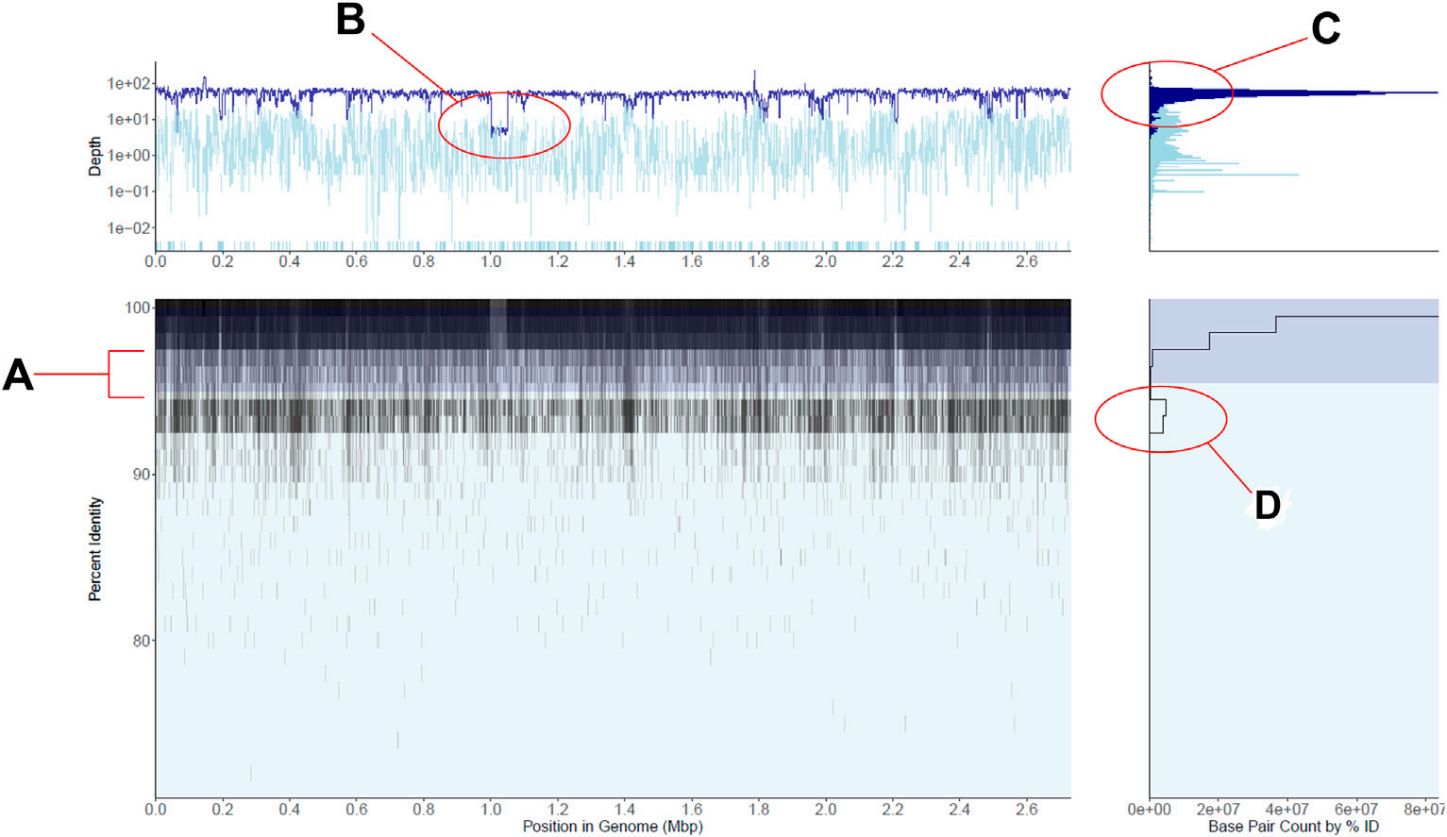
Recruitment plot displaying gut metagenomic reads mapped to a single *Staphylococcus aureus* reference genome. **(A)** is a 2-D histogram displaying the percent identity of reads to the reference genome on the y-axis and the position in the genome on the x-axis. Cell fill color darkens as more reads fall within the cell, i.e., the region of the genome and percent identity window the cell represents, in a logarithmic scale. Shaded in dark blue is a region indicates the plot’s current within-population percent nucleotide identity threshold, here shown at the tool’s default 95%. **(B)** is a line plot of the average depth of coverage per genome region on the main panel. The dark blue line displays depth of coverage for reads mapping to regions of the genome within the population threshold from panel **(A)**, and the light blue line displays depth for reads outside this population. Note the logarithmic scale in the base pair counts axis as well as the highlighted area of lower coverage, representing a reference genomic region not shared by the majority of the metagenomic population. **(C)** is a histogram of depths of coverage across the entire genome, with colors corresponding to within and outside-population as in panel. **(D)** is a histogram of the number of bases displayed in panel **(A)** (x-axis) which fall into particular percent identity windows (y-axis), here displayed in log scale. Note the second peak in the number of bases mapping in the 94–95% identity range, representing a co-occurring *S. aureus*-like population. See also main text for further details.

Metagenomics studies of natural microbial populations have recently revealed that bacteria and archaea predominantly form sequence-discrete populations with intra-population genomic sequence relatedness typically ranging from ∼95 to 100% genome-aggregate average nucleotide identity (or ANI) depending on the population considered (e.g., younger populations since the last population diversity sweep event show lower levels of intra-population diversity and thus, higher ANI). In contrast, ANI values between distinct populations are typically lower than 90% [Figure 1 and reviewed in (Caro-Quintero and Konstantinidis, 2012)]. Such sequence-discrete populations were recovered from many different habitats, including marine, freshwater, soils, sediment, human gut and biofilms, and were typically persistent over time and space [e.g., (Konstantinidis and DeLong 2008; Meziti et al., 2019; Olm et al., 2020)]. Therefore, these populations appear to be “species-like” and may constitute important units of microbial communities. This discovery is also important in medical microbiology; for instance, in identifying which population is the causative agent of disease (Pena-Gonzalez et al., 2019). More recent work has even shown that intermediate identity genotypes, for example, sharing 85–95% ANI, when present, are ecologically differentiated and thus, should probably be considered distinct species (Conrad et al., 2021; Rodriguez-R et al., 2021), rather than representing cultivation (or other sampling) biases (Murray et al., 2021).

Read recruitment plots are one of the most in-depth analyses to reveal and study sequence-discrete populations. In these plots, the reads of a metagenome are mapped against a genomic reference sequence that is representative of the population to be studied (e.g., an isolate genome or MAG). The mapping patterns that are revealed are informative about how well the metagenomic population matches the reference genome, gene content differences if any, the level of intra-population sequence diversity, and regions of sequence-discontinuity (Rusch et al., 2007; Konstantinidis and DeLong 2008) (Figure 1). Thus, read recruitment plots can provide a thorough and quantitative view of the natural population in a sample and its diversity, which represents highly useful information for several downstream analyses. Accordingly, several tools that can plot read mapping patterns have been developed for this purpose e.g., (Robinson et al., 2011; Zhu et al., 2013; Jaenicke et al., 2018). However, these tools typically provide no additional information or capabilities such as they do not include appropriate statistics to characterize the genome, gene allelic, and gene content diversity in spatial or time-series metagenomes and thus, do not allow targeted analyses of specific gene-based traits and exploration of selection pressure and population bottlenecks (Meziti et al., 2019).

Recently, we have developed bioinformatic scripts that can be applied to the read mapping output of a read recruitment plot to provide information based on read mapping that is not available by previous tools such as what is the average coverage of the genome by reads (a proxy for relative metagenome abundance), whether or not co-occurring populations exist in the dataset (sample) (Rodriguez and Konstantinidis, 2016), and which genes of the reference genome in the plot (isolate or MAG) are shared or not by the metagenomic population (Meziti, et al., 2019). Here, we present RecruitPlotEasy, a pipeline that integrates all these scripts into a single tool and represents a completely redesigned tool compared to that originally introduced as part of the enveomics script collection (Rodriguez and Konstantinidis, 2016) in order to scale-up with more data. RecruitPlotEasy also includes new, additional features such as the possibility to simultaneously view plots of multiple reference genomes and/or metagenomic read datasets and is interactive in that the user can browse over the plot to identify genes of interest and view their associated functional annotation (when provided) and relative abundance in the metagenome. Based on previous literature, we employed a (user-adjustable) 95% nucleotide identity threshold to identify reads that represent the same (target) population (a.k.a. *within* population diversity), while reads showing lower than 95% identity are considered to represent distinct, co-occurring populations (a.k.a. *outside* population). Using the RecruitPlotEasy tool requires no previous bioinformatics or coding skills.

## Installation and Inputs

RecruitPlotEasy is operated entirely through a graphical user interface (GUI) which manages the selection of inputs, the manipulation of data, and the creation of plots through simple buttons and drop-down menus. Further, all menus and options are annotated with tooltips and reports that help the user easily navigate the workflow of the Recruitment Plot without prior experience using the tool. RecruitPlotEasy is written in a pair of scripts, one in R and one in Python 3. This two-script design takes advantage of multiple visualization libraries and the GUI of the Shiny library available in R, while operating with modest computational resources enabled by Python.

To use the tool, the user needs only to download and install R, Rstudio, and then run a single command from the R terminal. This command installs any missing R dependencies, installs Miniconda if it is absent (to ensure that the right version of Python is subsequently installed), retrieves the R and Python functions, and launches the GUI for the user. While installation only occurs on the first use of RecruitPlotEasy, this same command is used in subsequent sessions to activate the GUI again. RecruitPlotEasy requires a user to supply one (or more) genome file(s) in FASTA format and at least one set of reads mapped to that genome file in either tabular BLAST or SAM/BAM formats. Users may optionally supply gene functional annotation in GFF for gene-level analysis.

## Methods

The graphical interface of RecruitPlotEasy opens in the user’s default web browser. This interface is organized into 4 tabs: Database Creation, Database Management, Recruitment Plot, and Interactive Plot (Supplementary Figure S8). The tabs organize the workflow of RecruitPlotEasy into smaller, manageable tasks where the options available on each page are directly relevant to the task that page supports. For instance, input selection occurs on the database creation tab, assessment of the contents of a database and the control of advanced options occurs on the database management tab, and the creation of plots occurs on the recruitment plot and interactive plot tabs. The workflow of the recruitment plot is further guided by multiple forms of user feedback. All buttons and input fields are annotated with tooltips that inform the user of the actions each button will perform upon hovering their cursor over it. This includes guidance on the type of file and the kind of data required in each input, and the consequences of changes made to plotting parameters. As inputs are selected, their formats are also checked for basic appropriateness.

The underlying data shown in a recruitment plot is a 2-dimensional matrix (or table) of counts. For a given genome, columns of this matrix correspond to successive regions of the genome and rows correspond to windows of percent nucleotide identity values. The width and height of each cell of the matrix are defined by the user, with defaults of 1000 base pairs for width and 0.5% identity for height. If viewing genes, percent identity windows are determined in the same way, but genome regions instead correspond exactly to the starts and ends of the gene sequences, with intergenic regions forming additional columns, as needed, to fill in the rest of the matrix. The cells of the matrix effectively form a 2-dimensional histogram of bins into which reads may fall.

To fill the matrix, reads aligning to the genome are assigned to their appropriate percent identity window and genome position bin. A user may choose to define percent identity to the reference as either the number of matches divided by the alignment length (local alignment) or matches divided by the entire length of the read, including unaligned sections (global alignment). After the percent identity row is determined, the read will increase the base pair (bp) count of the bin it falls into by its length. Should a read span two or more bins, each bin will receive its respective share of the read’s length according to exactly where the read mapped to the genome and the boundaries of the bins. The count for each bin represents the sum of all bases of all reads that map within the corresponding percent identity and region of the genome. Once every read has been processed, the filled matrix is passed to the plotting component of RecruitPlotEasy.

Reads may also be filtered based on minimum alignment length, minimum percent of the read aligning to the reference (not available for tabular BLAST), and by selecting only those reads which map best to the currently viewed genome (i.e. allowing each read to map only once across the set of genomes available in the database). Reads are not processed in any way prior to their selection from the database, meaning that any plot and any statistics or data export based on a plot are created only from the reads which pass the filtering parameters selected by the user (or the defaults when user makes no choices). A record of the exact query used to select reads from the current RecruitPlotEasy database is exported alongside any saved plot or data export in order to ensure that the reads used to generate a plot can be recovered at later dates.

## Output and Results

The recruitment plots from RecPlotEasy show four views of a single dataset; the main panel of a recruitment plot directly displays the underlying counts matrix, while the other three panels display useful summaries derived from the data in the main panel. Figure 1 shows an example of the read recruitment plot obtained with a single gut metagenome mapped against a *Staphylococcus aureus* reference genome. We consider the subplot in the bottom left the main panel, which shows how metagenomic reads of sufficient nucleotide identity and alignment (user defined) thresholds map against the reference genome. Because read recruitment plots commonly recruit tens of thousands to millions of reads from a metagenome it is computationally intractable to directly plot each read individually. Instead, we plot the sum of all bases from the reads that fall within a specific region of the main panel defined as a grid with cells consisting of base pair width (x-axis; default = 1000) and percent sequence identity of the read alignment height (y-axis; default = 0.5%) (Methods section above).

The panel on the bottom right shows the number of nucleotide bases (i.e., density of reads) at each unit of nucleotide identity (y-axis) in the main panel. Note that the specific case in Figure 1 shows an abundant *S. aureus* population in the sample, represented by a high density of high identity (>98% identity) reads mapping evenly across the reference genome along with a less abundant, closely related and co-occurring *S. aureus*-like population represented by the second lower peak in the number of mapped bases in the 94–95% identity range. The region between these two peaks shows the sequence discontinuity between the two sequence-discrete populations, one representing the reference genome and the other representing the sum of the remaining genomes.

Similarly, the upper left panel shows the coverage (i.e., how many times a base of the reference sequence is covered by mapped reads) of the genome at each unit of genomic position (x-axis) in the main panel. The darker blue color represents reads within the target population (default >95% nucleotide identity to the reference sequence) while the lighter blue represents lower identity reads considered outside the population (nucleotide threshold can be adjusted by the user). Note in Figure 1 that the top left panel shows fairly even read coverage across the genome for the target population with the exception of a few lower coverage regions while the outside of population coverage is more variable. This is an expected result for a single, homogenous population that is a good match to the reference genome. When no reads map to a genomic region (i.e., the region shows zero coverage), the region is displayed at the bottom of the panel for its respective group (dark blue target or light blue off-target), discontinuous from the rest of the line chart. Such low- or zero-coverage windows typically occur when a reference gene(s) is absent in the sampled population; browsing over the windows in the interactive mode can reveal which genes are found in these windows and their functional annotation, when the latter information is provided at the input stage. Hence, RecruitPlotEasy can reveal the gene content differences between the reference genome and the metagenomic population.

Finally, the top-right panel shows the histogram of coverage depth values over regions of the genome, which should reveal a tight distribution around the mean in cases where the reference genome represents a single population and a not-chimeric genome, like in the *S. aureus* example in Figure 1. A wider distribution would have been expected in the case of a chimeric genome that represents two or more populations with distinct *in-situ* abundances. For further details on the panels, see also Supplementary Figure S4. Supplementary Material includes additional methodological details; Supplementary Figures S5–S7 provide additional (less common) examples and use cases.

RecruitPlotEasy provides the user with the option to export their plots as high-quality figures and save the plotting data used to create them. Once the user has decided that they are satisfied with their plot as it appears in the GUI, they may provide a name and save the plotting data used to generate each recruitment plot sub-plot three in tab-delimited files and save a PDF of the plot image, laid out in a 16:9 aspect ratio to match the majority of modern screens. While RecruitPlotEasy uses the PDF format to save its plots, the graphics within each PDF are ultimately saved as an SVG that is both infinitely scalable without any loss in the figure’s resolution (i.e. it can be zoomed in on as much as desired without losing any clarity) and can be easily imported into common figure editing software that support SVG manipulation such as Adobe Illustrator for further editing, labelling, or other manipulations desired by a user.

The interactive plots created by RecruitPlotEasy may also be exported, but the files produced are quite different from the PDFs that are generated by the normal plotting approach. The R Plotly library is used to generate interactive recruitment plots, and these interactive plots are saved as HTML files that contain an independent copy of the data used to create the plot and the graphical results. These can be subsequently opened by most internet browsers, and do not require RecruitPlotEasy, R at all, or any other external software to be shared and viewed.

To better support integration in workflows, RecruitPlotEasy also includes a built-in read filtering function. When a database is built, RecrutPlotEasy makes a note of the location of each input read file and indexes reads by their name and the genome each mapped to. When viewing plots, reads at or above a user-defined percent identity cutoff may be added to a cart. By returning to the database management tab of the GUI, reads in the cart can be exported to filtered outputs in the same format as the inputs (although BAM is converted to SAM format) that will contain only those reads matching the selection criteria currently chosen by the user. Depending on the users’ chosen options, this can (and will, under default settings) include filtering reads to only their best matches, thus removing instances of reads mapping to multiple locations, filtering poorly aligned reads, selecting only reads mapping to specific genomes, and selecting only reads sufficiently similar to the reference sequence. This function is intended to be used in tandem with the plot saving component of RecruitPlotEasy: a user may inspect their read mapping results, tailor their filtering settings to match the properties of each genome as needed, and export reads alongside one or multiple recruitment plots that aid in justifying the choice of filtering parameters.

In summary, we have presented an advanced read recruitment plot tool that can provide a thorough and quantitative view of the natural population in a metagenome and its diversity, which represents highly useful information for several downstream analyses. Accordingly, we expect that RecruitPlotEasy will greatly facilitate microbiome research across the clinical and environmental fields and advance the toolbox for the analysis and summarization of large-scale genomic/metagenomic data and the communication of those results.

## Data Availability

Publicly available datasets were analyzed in this study. This data can be found here: https://github.com/KGerhardt/RecruitPlotEasy/tree/main/pub_data.
