# Supplementary figures and images for: RecruitPlotEasy: An Advanced Read Recruitment Plot Tool for Assessing Metagenomic Population Abundance and Genetic Diversity
Source: Front Bioinform. 2022 Jan 27;1:826701. doi: 10.3389/fbinf.2021.826701 (PMC9580866; doi:10.3389/fbinf.2021.826701)

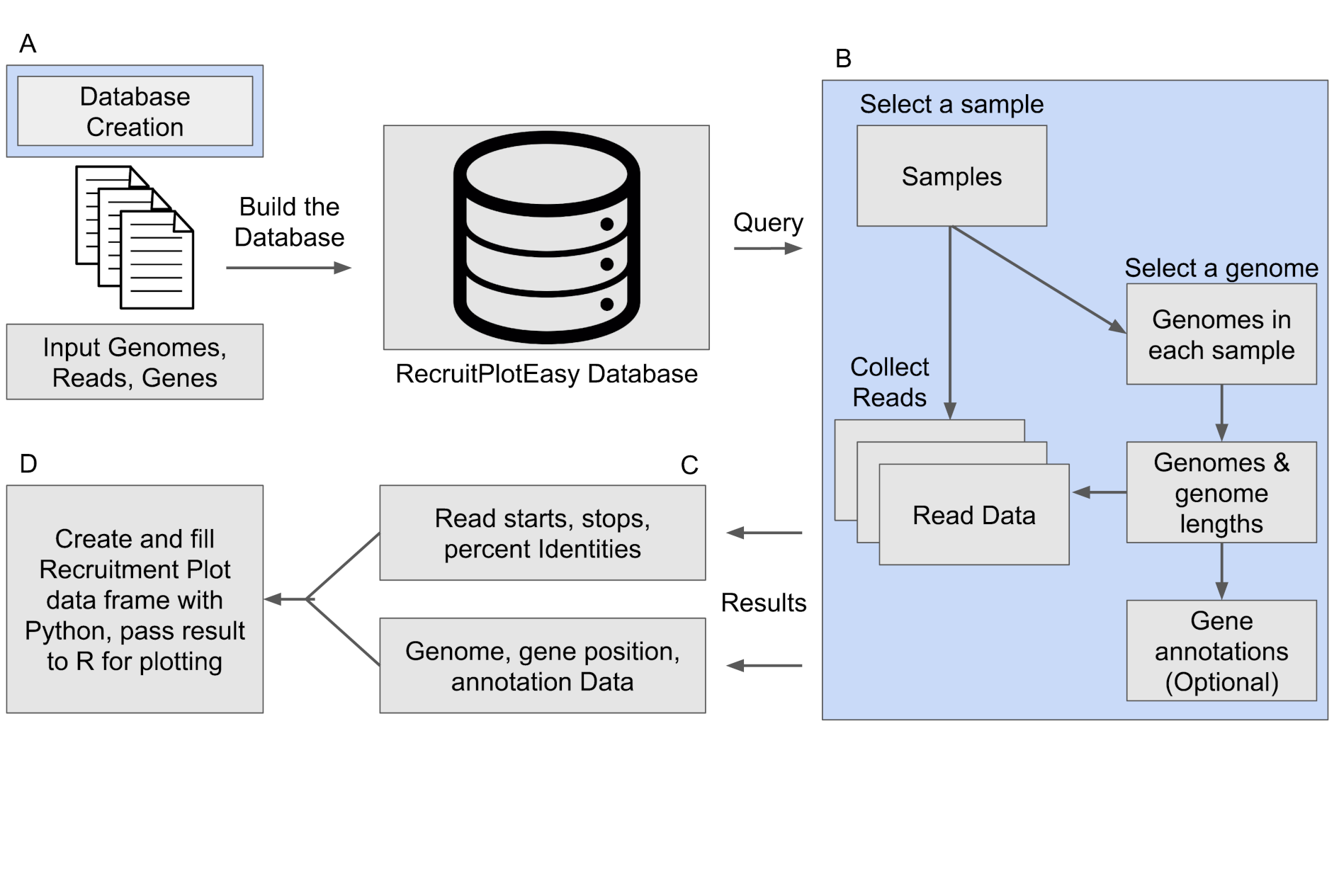

Supplement: Supplementary file 1 [file Image3.TIFF]

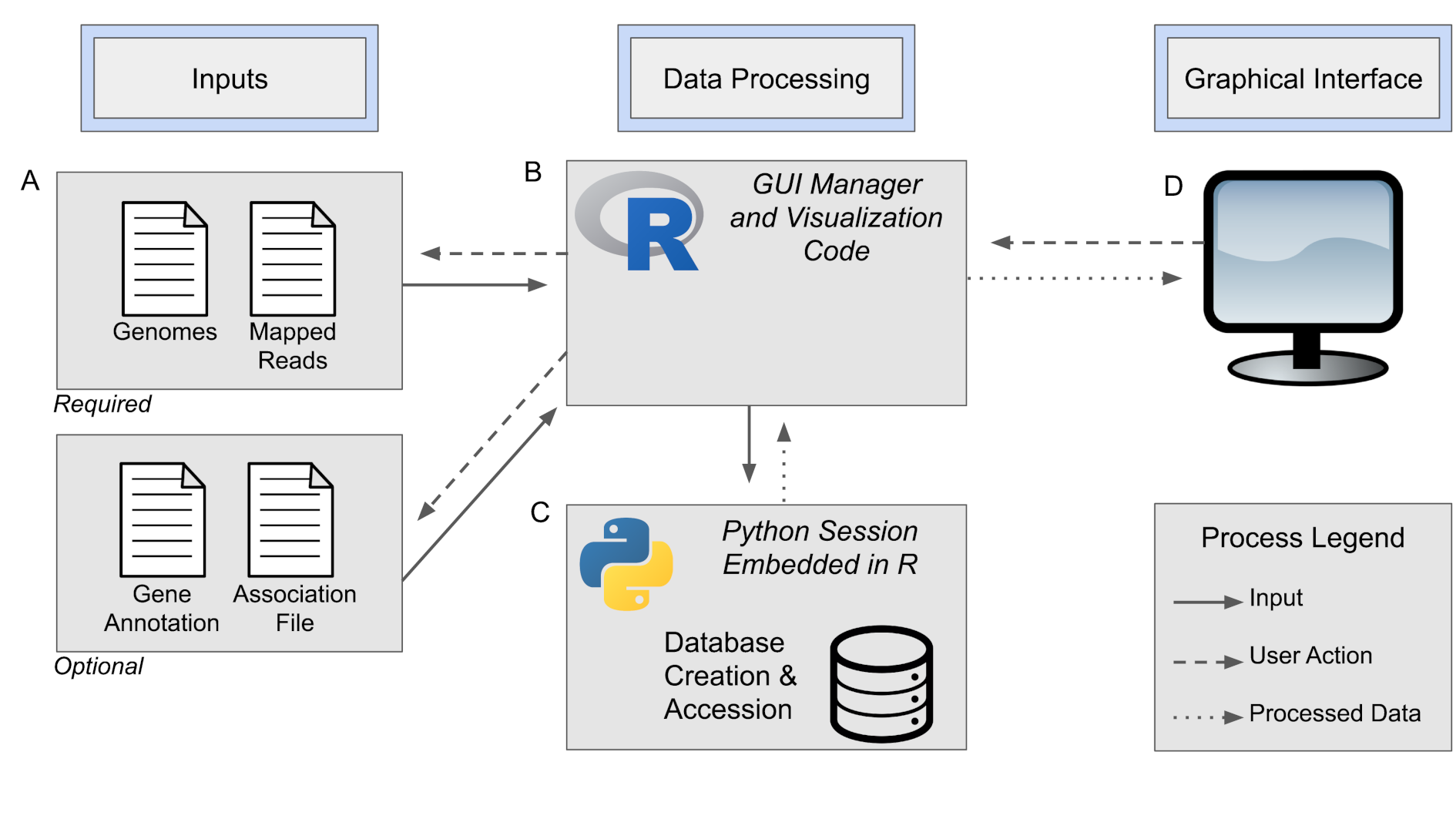

Supplement: Supplementary file 2 [file Image1.TIFF]

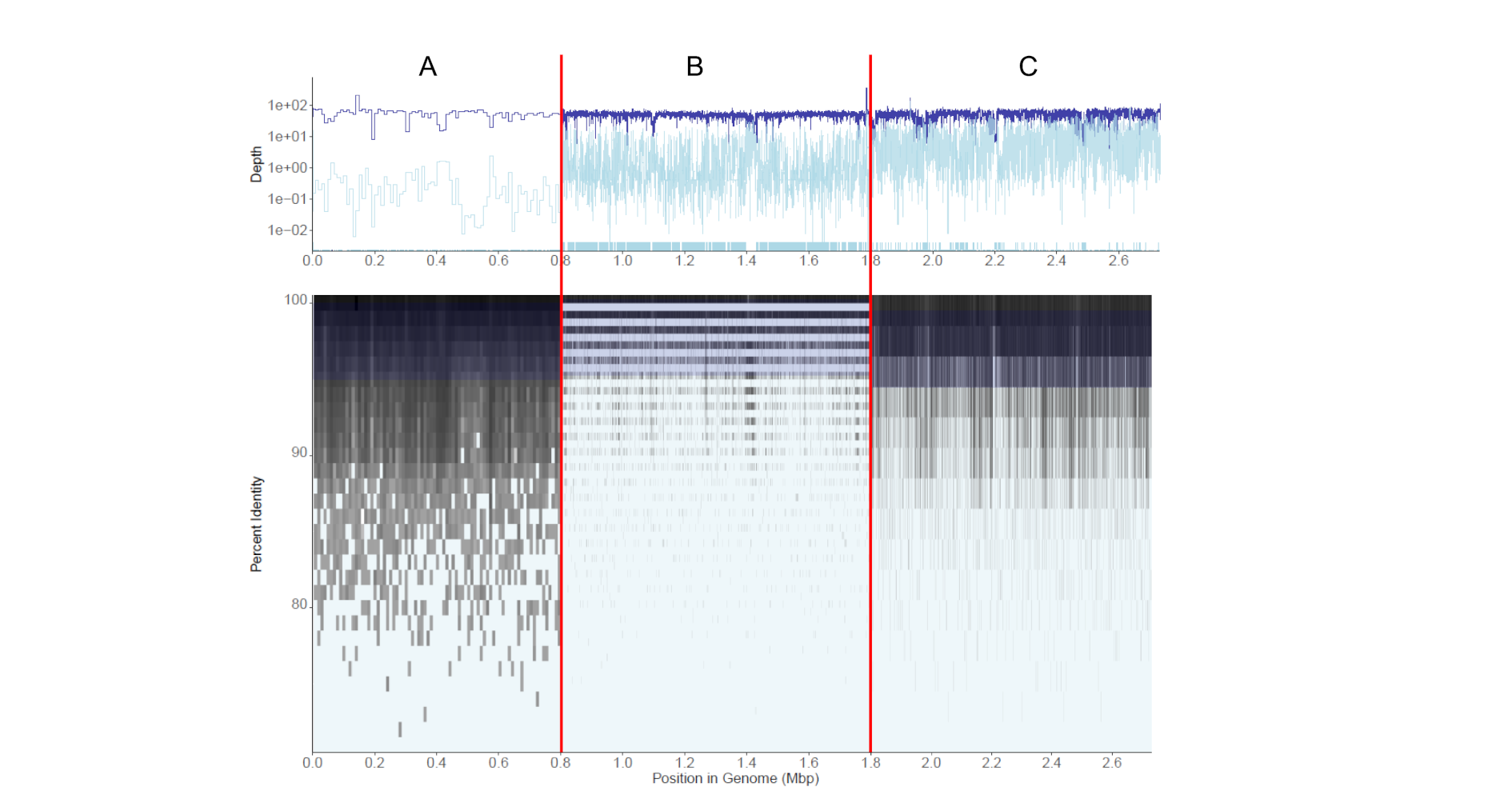

Supplement: Supplementary file 3 [file Image5.TIFF]

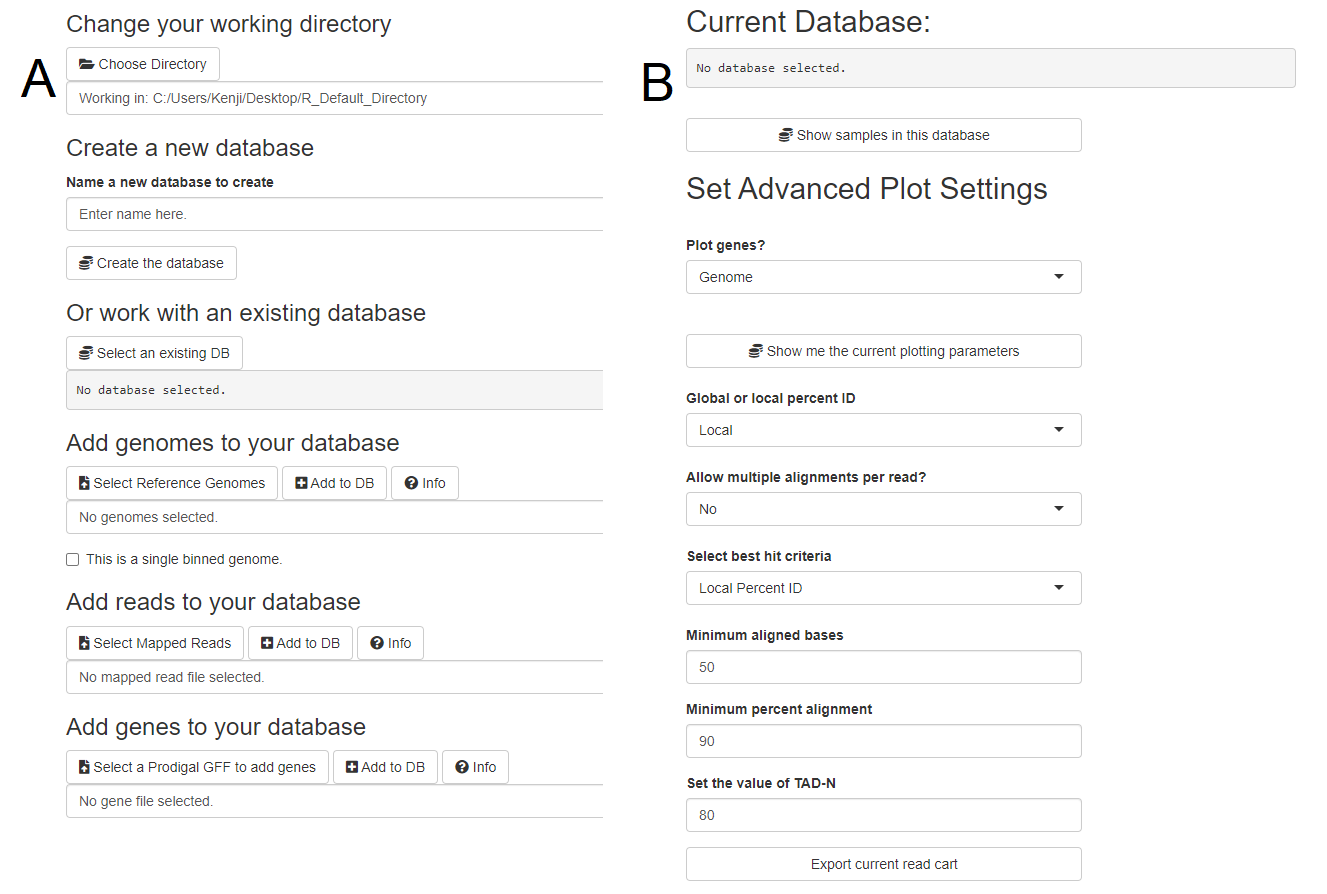

Supplement: Supplementary file 4 [file Image9.TIF]

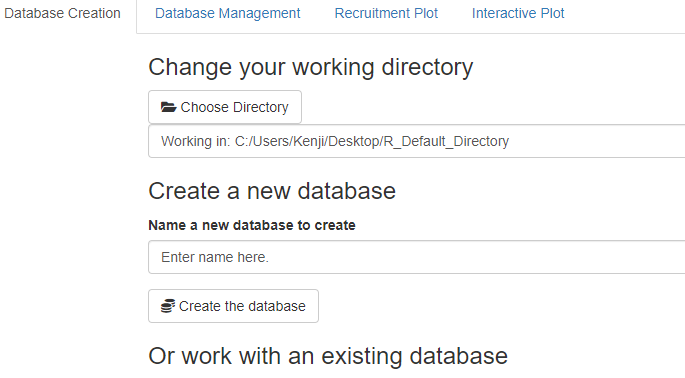

Supplement: Supplementary file 6 [file Image8.TIF]

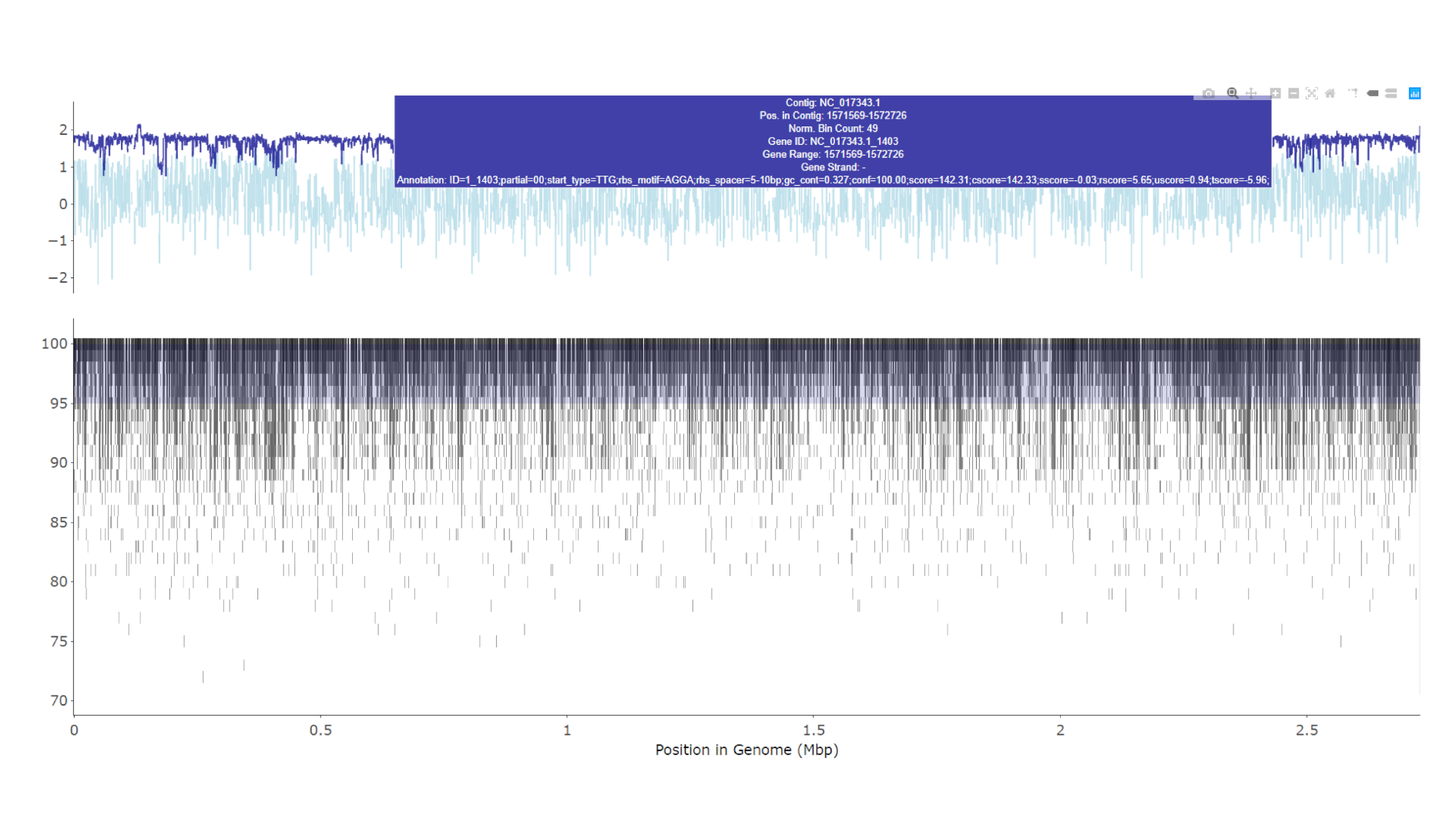

Supplement: Supplementary file 7 [file Image6.TIFF]

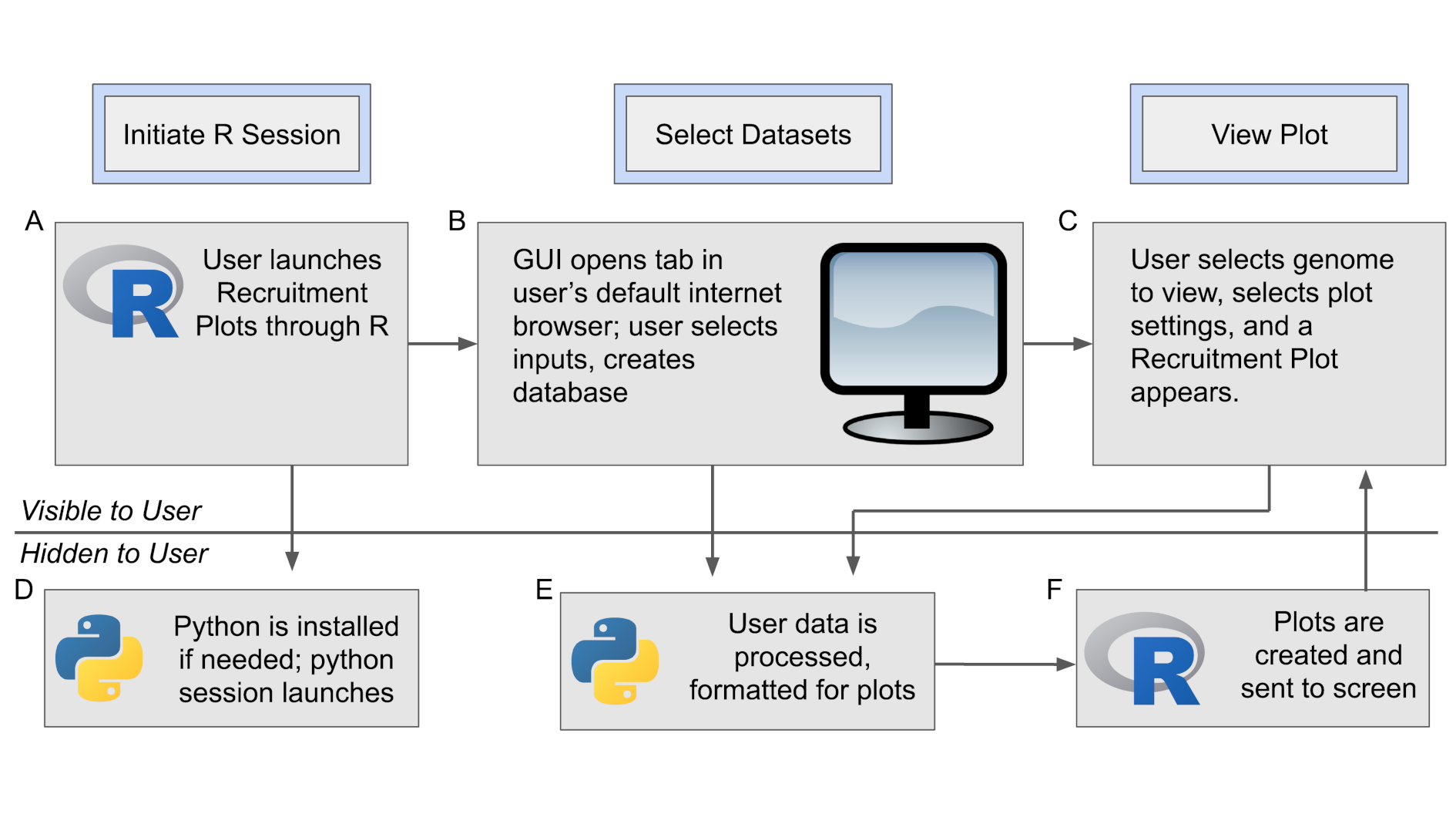

Supplement: Supplementary file 8 [file Image2.TIFF]

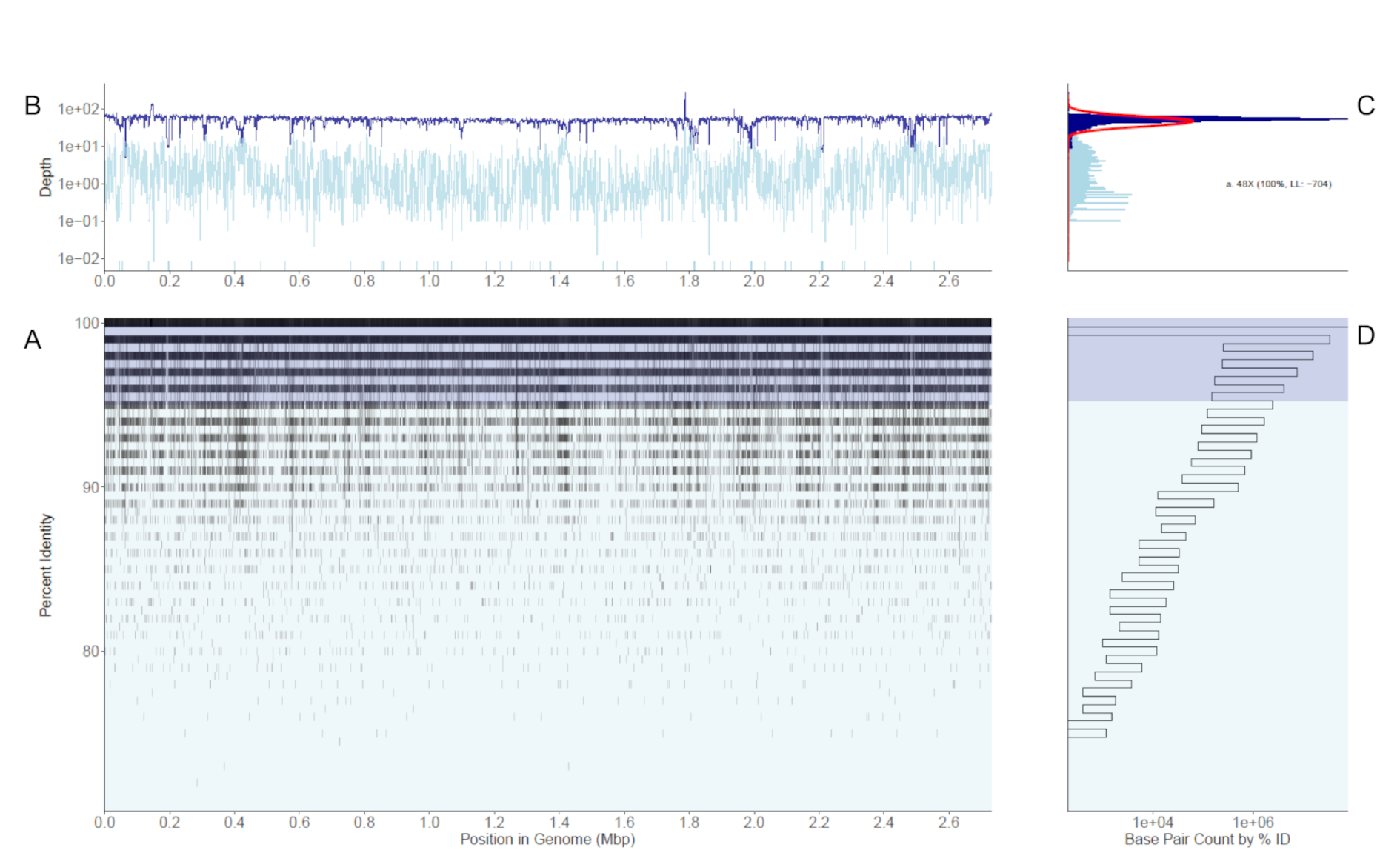

Supplement: Supplementary file 9 [file Image4.TIFF]

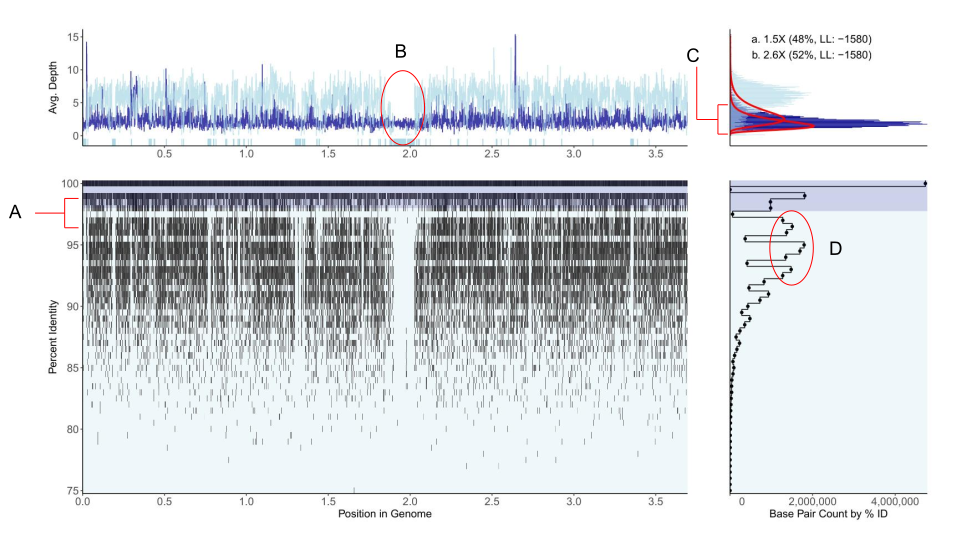

Supplement: Supplementary file 10 [file Image7.TIFF]
