# Supplementary material for: RecruitPlotEasy: An Advanced Read Recruitment Plot Tool for Assessing Metagenomic Population Abundance and Genetic Diversity
Source: Front Bioinform. 2022 Jan 27;1:826701. doi: 10.3389/fbinf.2021.826701 (PMC9580866; doi:10.3389/fbinf.2021.826701)
Supplement: Supplementary file 5 [file DataSheet1.PDF]

RecruitPlotEasy: An advanced read recruitment plot tool for assessing metagenomic population abundance and genetic diversity.

Kenji Gerhardt, Carlos A. Ruiz-Perez, Luis M. Rodriguez-R, Roth E. Conrad, and Konstantinos T. Konstantinidis

Supplementary Info

1 RecruitPlotEasy Implementation

Design Philosophy

RecruitPlotEasy's design is aimed at maximizing its accessibility to non-bioinformatician researchers seeking to perform genomic or metagenomic analyses. While the analysis provided by RecruitPlotEasy is useful to researchers of all levels of technical expertise, we worked to ensure that the tool is entirely usable by a researcher who is not familiar with the use of a command line and who has little to no experience in installing programs outside of the typical automated installers bundled with commercial software. To this end, RecruitPlotEasy is operated entirely through a graphical user interface (GUI) which manages the selection of inputs, the manipulation of data, and the creation of plots through simple buttons and drop-down menus. Further, all of these buttons and menus are accompanied by tooltips and reports that help the user navigate the workflow of RecruitPlotEasy without prior experience using the tool. All functionality contained within the RecruitPlotEasy scripts is designed to be operating system-independent and computational resource light so that the tool may be used on a personal computer.

Code Description

RecruitPlotEasy is written in a pair of scripts, one in R and one in Python 3. This two-script design seeks to take advantage of multiple visualization libraries and the GUI of the Shiny library available in R, while operating within modest computational resource limitations, enabled by Python. Typically, R scripts seeking to handle large quantities of data are heavily limited by the RAM available to a particular system; most operations in R load all data in a file into memory at once and thus cannot directly work on volumes of data larger than the computer memory available. By contrast, Python supports operating on data in a more resource efficient manner, meaning that any amount of data can be processed in small, fixed amounts of memory. As RecruitPlotEasy is a tool that intends to handle next-generation sequence (NGS) data on personal computers, the memory limitation present in R is a particularly salient concern. Consequently, RecruitPlotEasy uses its Python script to perform the primary data processing tasks of importing genomes and mapped reads, and summarizing results prior to plotting. This

allows the R portion of the code to operate only on comparatively small summaries of read mapping results, which can easily fit within the memory available to a typical personal laptop. The PyBam python module (https://github.com/luidale/pybam) is used in order to make BAM format mapped reads available for processing bto users on all operating systems

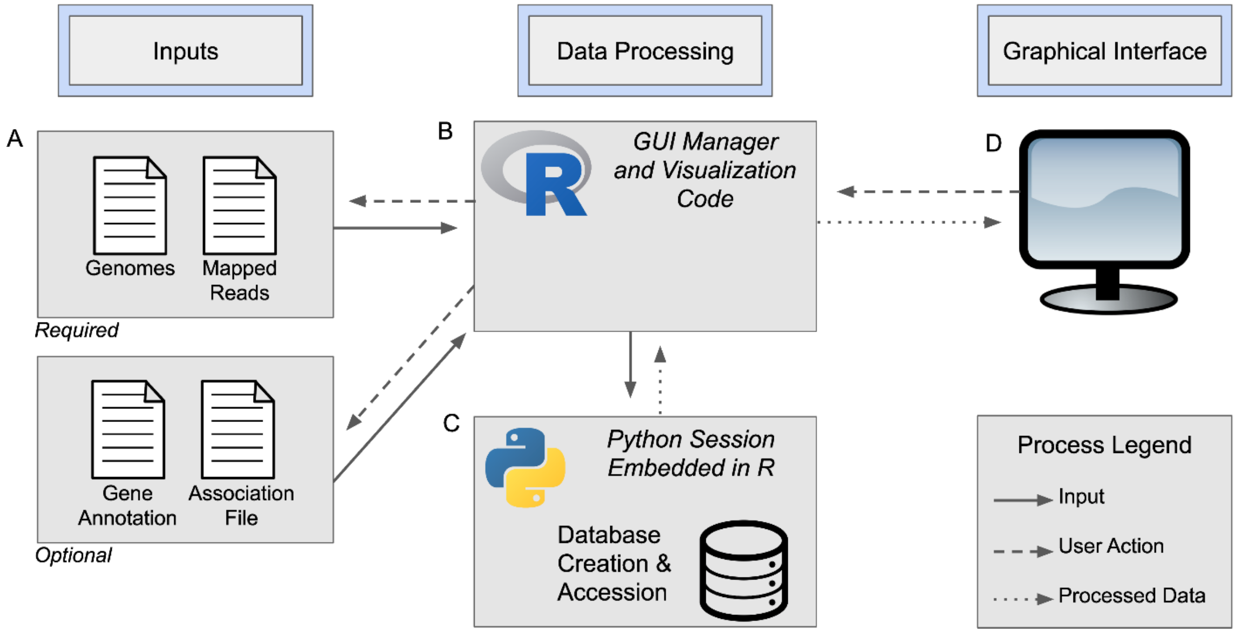

Supplemental Figure 1: **An overview of the RecruitPlotEasy workflow**. **A** shows input user data: FASTA format genomes or metagenome assembled genomes, short read data mapped to the selected reference genomes in either tabular BLAST or SAM/BAM format, Prodigal GFF format annotated genes, and the RecruitPlotEasy association file, which maps contigs onto MAGs in the case of binned assemblies. **B** shows the R layer of the RecruitPlotEasy program, where data management, command execution, and visualization occur. **C** shows the python layer, run on a python session embedded into the R session, where inputs are preprocessed, stored in a database, and formatted for visualization. **D** shows the interface between the user-end graphical interface and user-issued commands and the remainder of the RecruitPlotEasy program.

In order to ensure that neither the multi-script nor multi-language approach is an additional burden on the user, the R script of RecruitPlotEasy uses the Reticulate library to embed a Python session within the RecruitPlotEasy R session. This allows the R script to call Python functions as needed by RecruitPlotEasy rather than requiring external commands. RecruitPlotEasy checks for an existing conda installation or installs Miniconda within an R-specific directory if an existing installation is not found, then creates an environment for installing its supporting R and Python libraries. This ensures that the correct version of Python

will be available to RecruitPlotEasy even if a user did not previously have any Python installation on their system. In addition, the creation of a new environment insulates any existing Anaconda installations from RecruitPlotEasy. The creation of the RecruitPlotEasy environment, and installation of Miniconda if necessary, is done once during the first run of the RecruitPlotEasy on the user's system, and the environment is subsequently reused by later RecruitPlotEasy sessions on the same system.

Fully installing RecruitPlotEasy requires R, RStudio, Python, NumPy, Miniconda, and multiple R libraries. The installation of R and RStudio must be performed by a user, while the other dependencies can be taken care of through RecruitPlotEasy's library installation and GUI initialization functionality. In total, a user should expect all of this to require about 800MB of disk space. During use, RecruitPlotEasy consumes a baseline of approximately 500MB of RAM and additional RAM depending on the visualizations produced by the tool. Visualization of a 3.6 Mbp genome under the default settings of 0.5% identity windows and 1000bp genome windows as both a static and an interactive plot requires approximately 300MB of RAM in addition to the 500MB at launch for a total of approximately 800MB. Visualization of a 10.8Mbp genome under the same settings requires an additional 500MB of RAM for a total of 1GB of RAM. Therefore, most laptops will be more than capable of visualizing any prokaryotic genome.

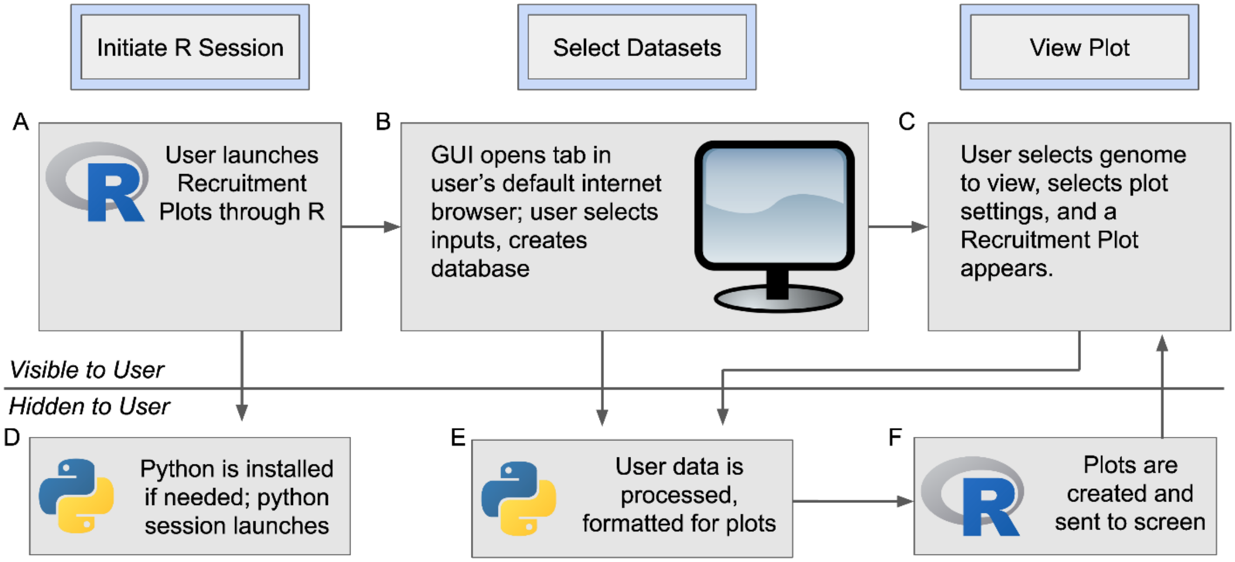

Supplemental Figure 2: **An overview of the use of RecruitPlotEasy from the user perspective**. **A**, **B**, and **C** display the user-side experience of using RecruitPlotEasy, and **D**, **E**, and **F** show the underlying processes that their choices interact with. Users launch the tool through R as shown in panel **A**. The program checks for its dependencies and installs them if needed in panel **D**, with the installation only performed on first use. Once dependencies are present, the GUI activates as shown in panel **B** and database creation takes place from user inputs. **C**, **E**, and **F** then interact in a cycle where each new recruitment plot is generated from

the database and processed with R before being seen by the user.

Database Design

The primary aim of RecruitPlotEasy is to produce recruitment plots of a user's mapped read data in an interactive manner. Interactivity demands that a user be able to flexibly control their data, rapidly produce results, and browse their results easily. RecruitPlotEasy provides users control over their data by making it easy to select which genome is shown and dictate the parameters of their recruitment plots through simple menus and sliders. However, providing this degree of control means that RecruitPlotEasy must wait for a user to supply these parameters before it can begin processing the user's data for plotting. This places an additional burden on the temporal aspect of interactivity, as a user must wait for results for as long as RecruitPlotEasy takes to create a user's requested recruitment plot. To maintain interactivity, the duration a user is kept waiting for their plot must be as brief as possible.

As NGS datasets tend to be large, rapidly producing plots means controlling the data effectively. RecruitPlotEasy achieves this by producing an indexed SQL database as an intermediate between the user's inputs and the plotting function of RecruitPlotEasy (Supplemental Figure 3). A RecruitPlotEasy database stores only the information required to make a recruitment plot, ensures the format of data is consistent, and pre-calculates percent identity values if necessary, so that they do not have to be calculated at the time a user wishes to create a plot. The indexes of the database further allow RecruitPlotEasy to directly access all and only the data required to create a particular recruitment plot, so that no work extraneous to the current plot is done by the program.

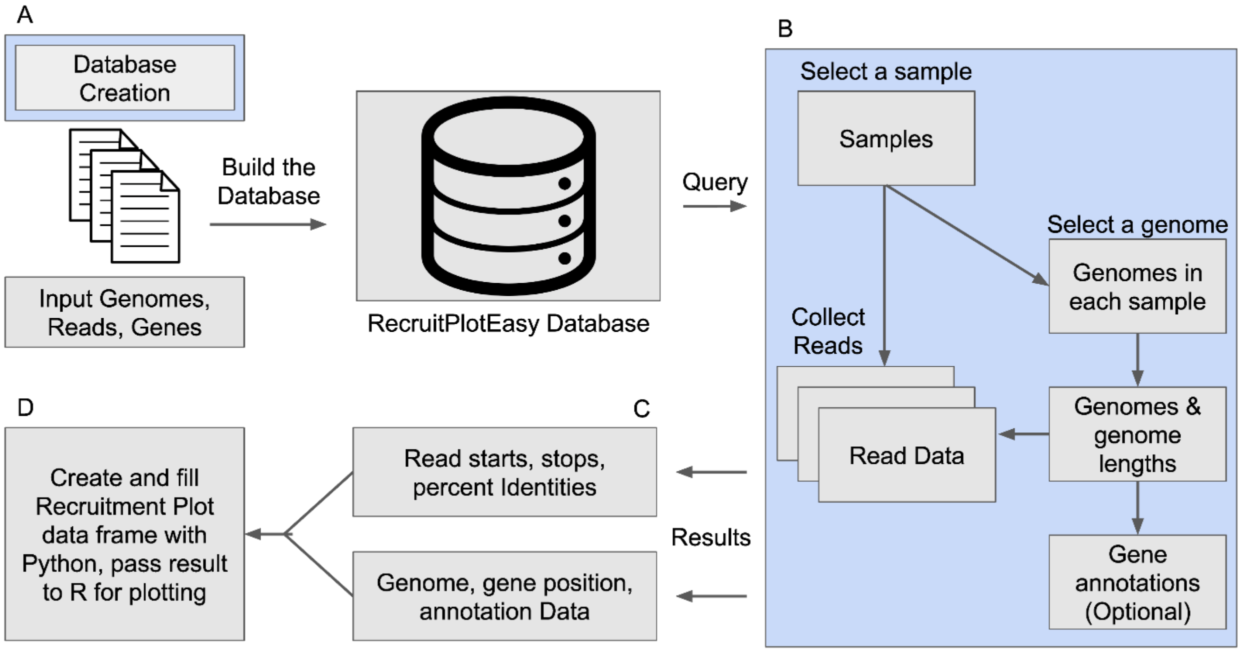

Supplemental Figure 3: **A diagram of the RecruitPlotEasy database and query process**. **A** shows the initial creation of the database from its inputs. **B** shows a sample query and the internal structure and relations of the database. Users select a sample and a genome and the database returns **C** information about the genome, reads in the sample mapping to the genome, and gene data if requested. **D** the results of the query are formatted for plotting and passed to R.

How to Read a Recruitment Plot

The recruitment plots created by RecruitPlotEasy show 4 views of a single dataset. The main panel of a recruitment plot directly displays the underlying data while the other three plots display useful summaries derived from the data in the main panel. To read a recruitment plot, a user needs to understand what is being shown in the main panel and how this data is summarized in the other 3 plots.

The underlying data shown in a recruitment plot is a 2-dimensional matrix (or table) of counts. For a given genome, columns of this matrix correspond to successive regions of the genome and rows correspond to windows of percent nucleotide identity values. The width and height of each cell of the matrix are defined by the user, with defaults of 1000 base pairs for width and 0.5% identity for height. If viewing genes, percent identity windows are determined in the same way, but genome regions instead correspond exactly to the starts and ends of the gene sequences, with intergenic regions forming additional columns, as needed, to fill in the rest of the matrix. The cells of the matrix effectively form a 2-dimensional histogram of bins into which reads may fall. The count for each bin represents the sum of all bases of all reads that map within the corresponding percent identity and region of the genome.

The percent identity of each read is calculated by dividing the number of bases matching the reference sequence by the length of the read, and all bases from the read will be assigned to the corresponding percent identity window. After the percent identity row is determined, the read will increase the base pair (bp) count of the bin it falls into by its length. Should a read span two or more bins, each bin will receive its respective share of the read’s length according to exactly where the read mapped to the genome and the boundaries of the bins. Once every read has been processed, the filled matrix is passed to the plotting component of RecruitPlotEasy.

The main panel (Supplemental Figure 4A) of a recruitment plot is located in the lower-left corner. This panel is similar to the traditional read recruitment plot (Konstantinidis and DeLong, 2008; Rusch, et al., 2007), but displays the count matrix created by the reads as a two-dimensional histogram rather than directly plotting the reads themselves. This allows RecruitPlotEasy to accommodate the millions of reads provided by short-read (e.g., Illumina) shotgun metagenomes with minimal computational requirements. The plot is a grid of cells, which show the count of base pairs falling into the corresponding bins of the counts matrix. The X-axis (i.e., position across the genome) is shared by the top-left plot and the Y-axis (i.e., percent identity window) is shared by the lower-right plot, including the boundaries dividing the genome into regions and percent identity windows, respectively. Darker cells on the plot indicate more

reads fell into these cells, and a complete absence of color in a cell indicates that no read mapped to that region, at that level of percent identity. Overlaid on the main and lower-right panels is a user-defined shaded blue region, covering 95% to 100% identity by default. This shaded region indicates the threshold to consider reads mapping to the genome as part of the same population (or group), and is used to separate the user's reads into a within-population (shaded region on the lower plots; dark blue on the upper plots) and an outside-population (outside the shaded region on the lower plots; light blue on the upper plots). This division into within- vs. outside the population has no consequence for reading the lower two plots but is shown for visual reference. On the upper plots, the values are computed separately for members of these two populations.

The bottom right panel (Supplemental Figure 4D) is a one-dimensional histogram of the count of base pairs falling into each percent identity window, without stratification by their position in the genome as shown in the main panel. The same within- vs. outside the population shading displayed on the main panel is replicated here; an effective within-population threshold will capture the overwhelming majority of aligned bases within the population, which is much easier to see on this plot than in the main panel.

The top-left panel (Supplemental Figure 4B) is a dual line chart displaying the average depth of sequencing coverage for each region of the genome. The dark blue line corresponds to reads falling within the population (inside the blue shaded region) displayed in the main panel, and the light blue line corresponds to reads falling outside the population (outside the shaded region). For each region of the genome, the count of bases falling into the percent identity within- and outside-population are summed separately, then averaged over the length of the corresponding region to produce the depth of coverage. If no reads mapped in a particular region of the genome at any of the percent identity windows in either the within- or outside-population (i.e., the region shows zero coverage), the region is displayed at the bottom of the panel for its respective group, discontinuous from the rest of the line chart. Particularly when viewing genes, low or absent read alignment to specific genes can quickly identify gene content differences between the sampled (metagenomic) population and the reference genome used in the recruitment plot, and their annotations can be viewed in the interactive variant of the recruitment plot.

The top-right panel (Supplemental Figure 4C) is a summary of the sequencing depth chart on the top-left, rather than a direct summary of the main panel. This plot contains two histograms mapping to the within- and outside-population of the sequencing depth chart (again shown in dark blue and light blue, respectively), and shows the distribution of depths of coverage for each region of the genome, in each population of reads. In a sample capturing a single population and mapping to a non-contaminated, high-quality reference genome sequence, the within-population of reads should form a single normal distribution centered around the average sequencing depth of the entire genome. However, samples of mixed populations, or a poorly selected in-group threshold, can produce multiple peaks at different depths of coverage. RecruitPlotEasy has a module that fits a gaussian mixed model to the within-population distribution and reports any significant peaks it discovers, with peaks beyond the first/dominant

peak corresponding to significant subpopulations detected in the sample, as described in Rodriguez-R and Konstantinidis, PeerJ preprints, 2016.

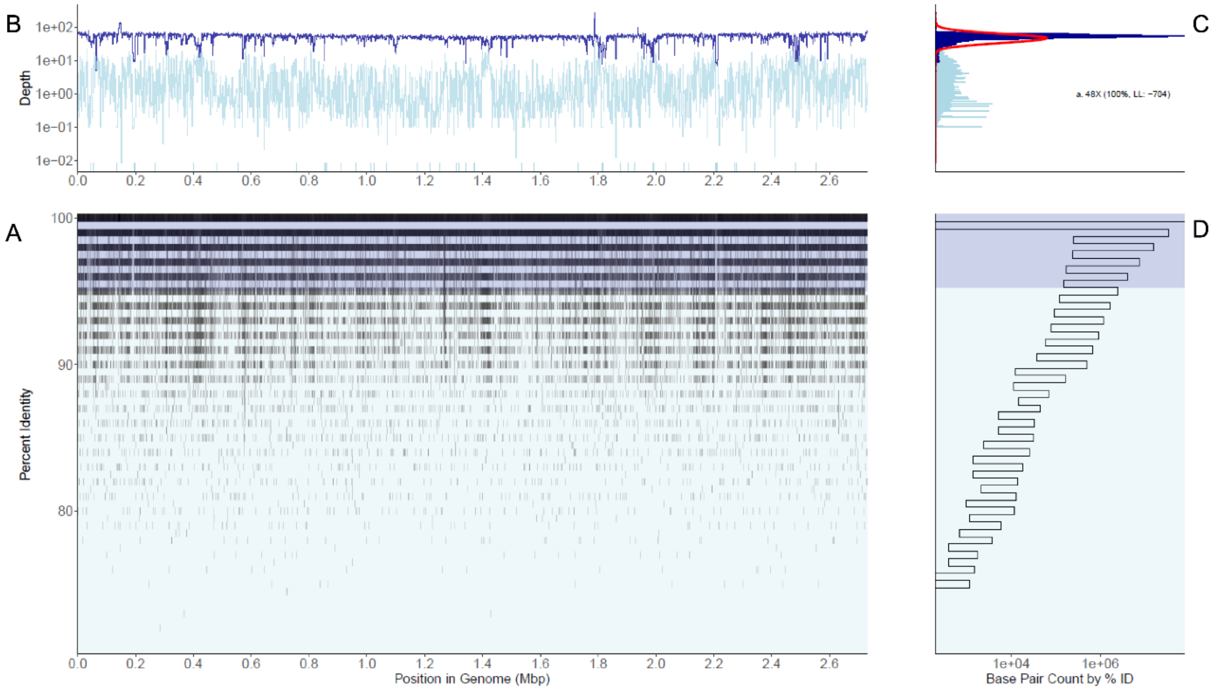

Supplemental Figure 4: **An example Recruitment Plot, displaying reads mapped to a single *Staphylococcus aureus* genome**. **A** is a 2-D histogram displaying the percent identity of reads to the reference genome on the Y-axis and the position in the genome on the X-axis. Cell fill color darkens as more reads fall within the cell, i.e., the region of the genome and percent identity window the cell represents, in a logarithmic scale. Shaded in dark blue is a region indicating the plot's current within-population percent identity threshold, here shown at the tool's default 95%. **B** is a line plot of the average depth of coverage per genome region on the main panel. The dark blue line displays depth of coverage for reads mapping to regions of the genome within the population threshold from panel **A**, and the light blue line displays depth for reads outside this population. Note the logarithmic scale in the base pair counts axis. **C** is a histogram of depths of coverage across the entire genome, with colors corresponding to within and outside-population as in panel **B**. The associated red overlay on the top-right panel is the result of a Gaussian mixed model used to determine distinct groupings of depth of coverage values across the genome and provides reports of the genome-average depth of coverage and the associated weight for each grouping. **D** is a histogram of the number of the number of bases displayed in panel **A** which fall into particular percent identity windows, here displayed in log scale.

Plotting Parameters

RecruitPlotEasy's plots and internal statistics are controlled by a collection of parameters, all of which can be modified by the user to suit their particular data and questions. Several of the

parameters must be set prior to loading data for plotting, and several can be changed to affect plots interactively. All parameters are presented in sidebars on the two plotting tabs of RecruitPlotEasy so that they are easy to modify as plotting occurs, and are shared between the static and interactive plotting tabs and data outputs; this ensures that presentations of the data are consistent with each other.

A user may control the basic resolution of a recruitment plot by setting the number of base pairs and the range of percent identity values that make up each cell on the main plot, both of which are reflected in the granularity of the plots (Supplemental figure 5) and the data underwriting them. By default, the regions of the genome are 1000 base pairs (bp) wide and the percent identity bins are 0.5 percent identity high. These choices are optimized for the properties of short read data and the distribution of bacterial gene lengths; a user may change these parameters for different types of data. As the percent identity of a read can only vary by whole bases, it is often impossible for short read data to be meaningfully resolved beyond 1 percent identity to the reference; increasing the resolution further typically results only in meaningless computation and harder-to-read plots for short reads (but not for long reads of 500-1000bp or longer). The choice of 1000 base pairs as the default bin width reflects the most common length of bacterial genes. The length of the genome window option is removed in the case of plotting genes, as RecruitPlotEasy instead creates windows exactly mapping onto gene starts and ends, and is replaced by the choice to view only genes, only intergenic regions, or all regions of the genome.

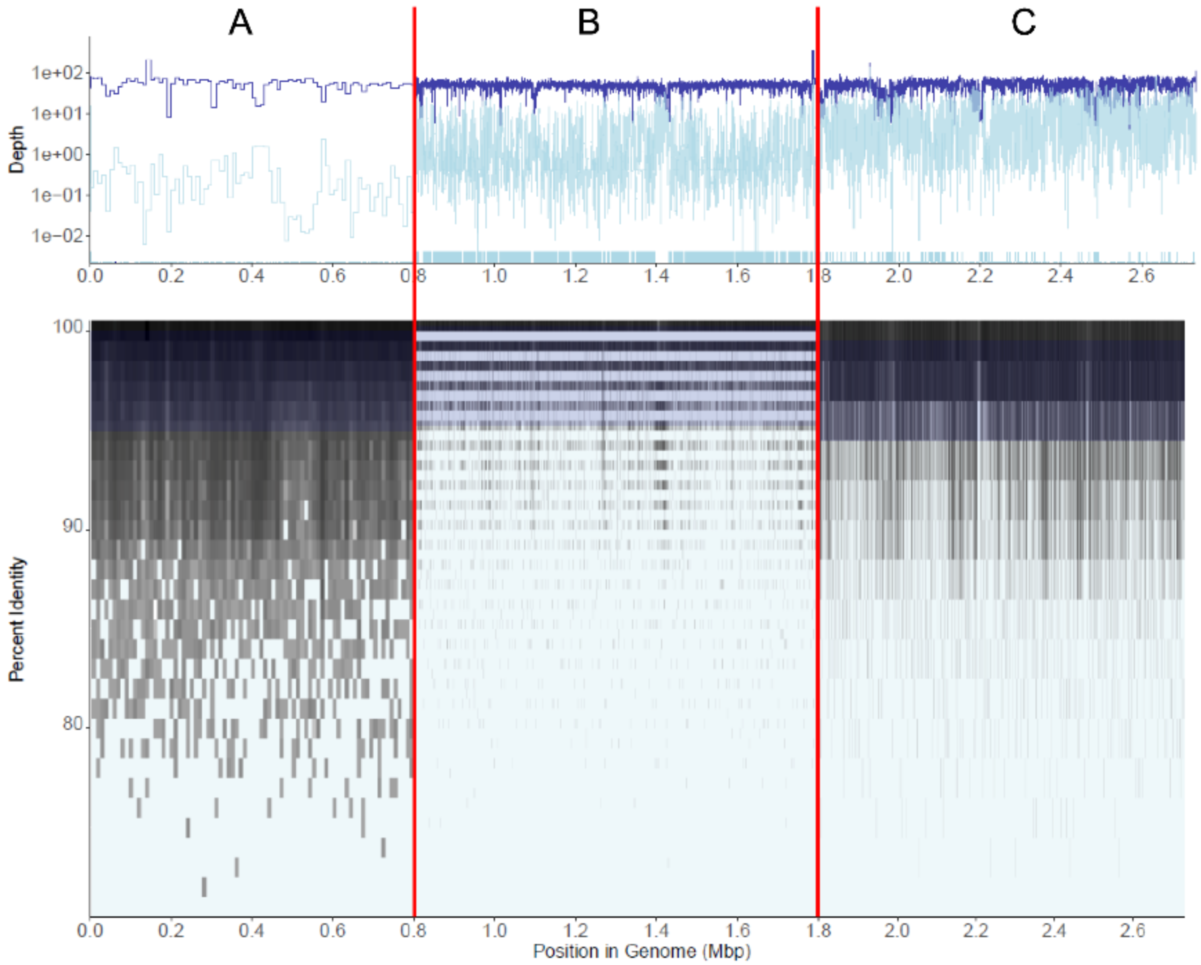

Supplemental figure 5: **Sections of a single recruitment plot displayed under three different pairs of genome width and percent identity height parameters. A** shows the genome resolved with regions 10 kbp-long and percent identity windows of 1%. **B** shows the default recruitment plot settings of 1 kbp-long genome regions and percent identity windows of 0.5%. **C** shows a higher genome resolution with 250 bp regions and 2% identity windows. Note in **C** that the 95% within-population threshold results in the 94-96% identity window being part of the outside-population instead of the within-population; this results in a higher outside-population depth of coverage and lower within-population depth of coverage (imperceptible in the lower graph but noticeable in the upper graph, light blue part).

The remaining plotting options available to the user affect the lower identity threshold of the within-population for RecruitPlotEasy's depth statistics, whether the counts of base pairs at each percent identity are displayed in linear or logarithmic units, and whether RecruitPlotEasy will attempt to identify distinct sub-populations in terms of their depth coverage values within the current within-population of reads. The latter setting basically determines whether or not the code for finding one or more distinct peaks of depth of coverage values within the population will be executed to produce the histogram shown on the upper right panel of Supplementary

Figure 3 (Panel C). The default is off for computational efficiency, and it is recommended that the users turn this setting on once they are satisfied with the main panel of the recruitment plot. The within-population default settings of a recruitment plot are intended to separate (define) a sequence-discrete population and assess the depth of coverage and gene content differences separately from spuriously mapped reads or closely related populations based on the intra-diversity most commonly observed for natural sequence-discrete populations as described previously (Caro-Quintero and Konstantinidis EMI 2012).

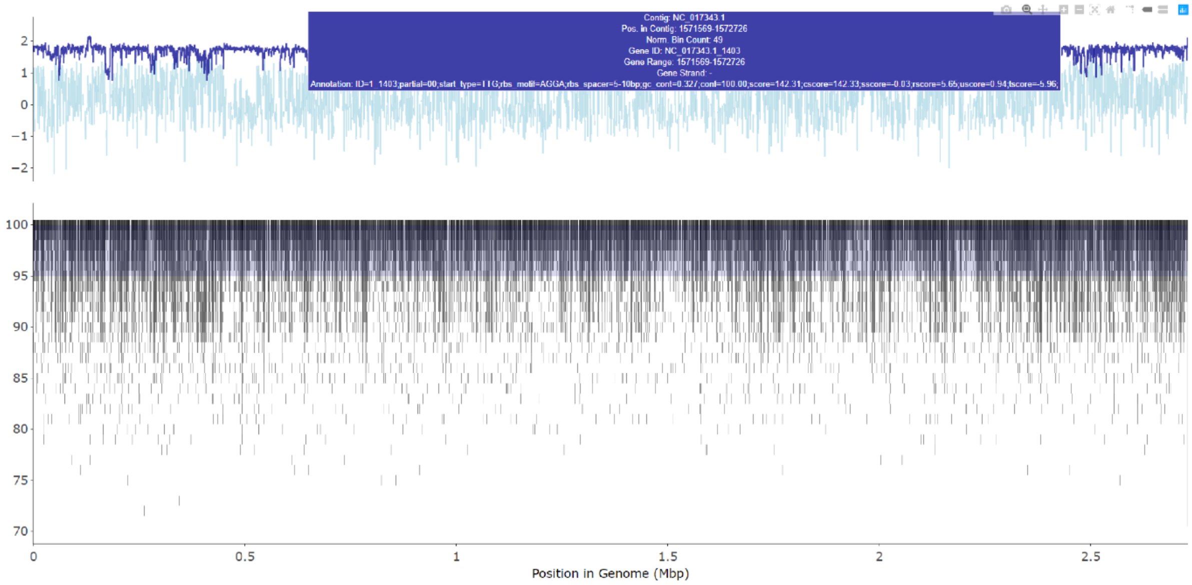

Supplemental figure 6: **A recruitment plot showing the annotation information of a gene on the sequencing depth chart (top) after the selection of the gene by the user.** Included are the name of the contig, the name of the gene, the location of the gene in the contig, the normalized read depth over the gene, the coding strand, and additional annotation information. Note that both position in contig and gene range are reported - position in contig may be slightly truncated in cases where genes overlap.

Recruitment Plot Use Case

A recruitment plot allows a user to detect and separate reads originating from populations that are difficult to distinguish at the genomic level. To exemplify this capacity, we simulated reads from the six Burkholderia genomes described in Konstantinidis, Tiedje et. Al, 2007, which share whole genome ANI values ranging between 84.7% and 99.9%. 50 thousand reads were generated for each genome using Bbtools' randomreads module (Bushnell et. al, 2017) under default settings. We selected one genome as a target sequence, *Burkholderia lata* (ENA accession GCA_000012945.1), and mapped reads originating from all of the genomes back to it using Bowtie2 (Langmead et. al, 2012) using the –sensitive-local flag. This scenario emulates an assembly and mapping effort for an environment in which several closely related populations are present, but only one genome has been successfully assembled. The genomes used and a RecruitPlotEasy database created from the reads generated in this use-case are available on the RecruitPlotEasy GitHub.

In this example, 178,324 reads of the possible 300,000 mapped to the target sequence. All 50,000 reads originating from the reference sequence successfully mapped back to it at or above 94.4% identity, while 128,324 reads originating from the other 5 genomes spuriously mapped at percent identities ranging from 20.0% to 100%. Filtering this readmapping effort at 95% identity to the reference captures 49,997 of the reads originating from the target genome; however, this filter also captures 34,167 spuriously mapped reads, meaning that only 59.4% of the reads that pass this 95% identity filter actually belong to the correct genome.

Using RecruitPlotEasy to assess this data (Fig. S7 B) shows a concentration of reads mapped near 100% identity alongside a second swell of reads that appear to be distributed around 94% identity to the reference sequence. A local minimum can be seen on the plot at 97.5% identity, significantly above than the 95% cutoff typical in many analyses. Selecting 97.5% identity as the cutoff as indicated by the plot results in capturing 49,678 (99.3%) of the on-target reads while reducing the spuriously mapped reads retained to only 7646 (22.3%) when compared with filtering at 95%, improving the selection of correctly filtered reads to 86.7%. A slightly more conservative cutoff of 98% identity retains 97% of on-target reads, only 11.9% of spurious reads, and results in 92.2% of reads passing the filter being correctly identified as originating from the reference genome. Alternatively, one could adjust the nucleotide identity threshold to about 90% in order to capture (the reads from) all these related *Burkholderia* genomes as a single, diverse population, if this fits the research questions better (for tracking the distinct, sequence-discrete population represented by the reference *Burkholderia* genome, the 98% threshold seems to be more appropriate in this case, as explained above).

Additionally, the presence of a concentration of reads below 100% identity reveals to the user that there is likely at least one genome present in the environment which has not been successfully assembled, and provides a clue as to how dissimilar that genome is to the reference sequence. Further inspection shows several regions of the genome where reads below 98%

identity infrequently map, possibly revealing genes, operons, or a plasmid present only in the target sequence and not in closely related populations (Fig S7 A).

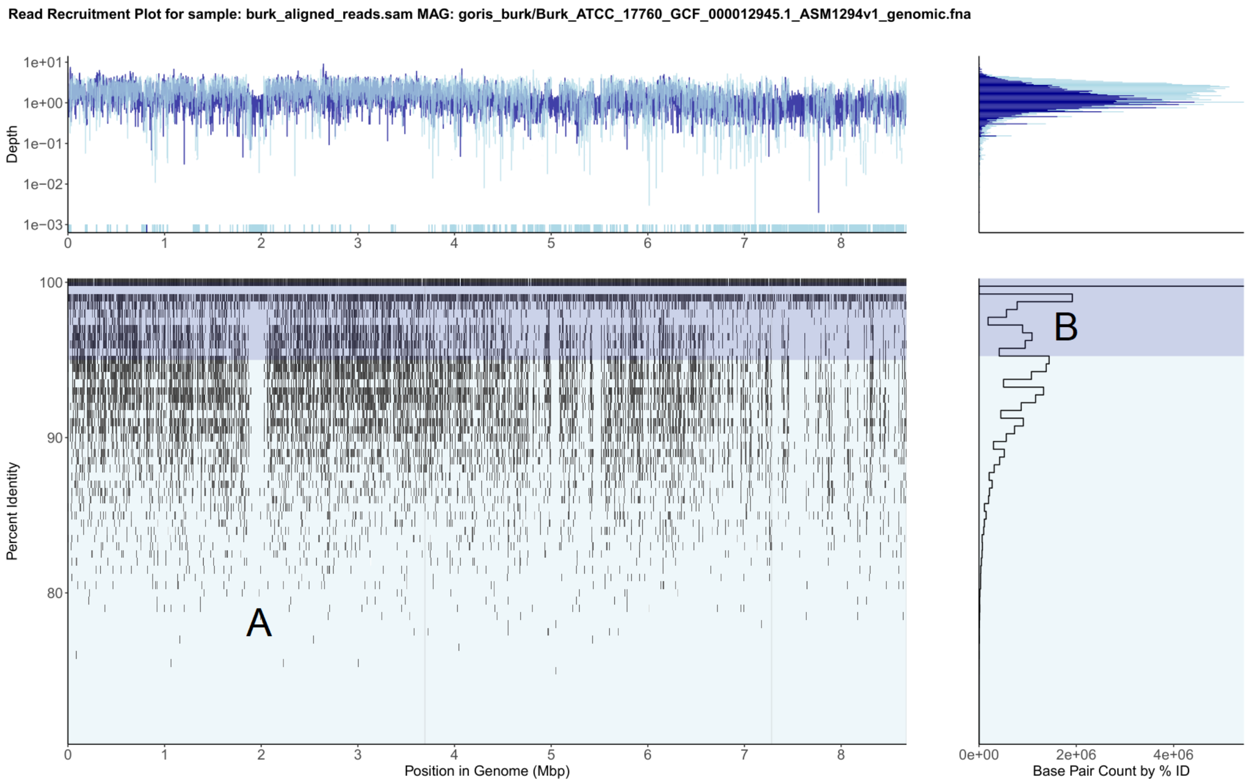

Supplemental figure 7: **A Recruitment Plot displaying simulated reads mapped against *Burkholderia lata*.** Reads simulated from six *Burkholderia* genomes were mapped against a single *B. lata* genome to demonstrate the inappropriateness of a 95% read mapping identity cutoff in some cases. (A) A region of significant drop in read mapping at lower percent identities, indicating features present in the reference *Burkholderia* absent in related species. (B) A local minimum in read coverage at 97.5% identity to the reference before a significant increase at lower percent identities. The read of lower identities typically indicate a closely related species present in a sample whose genome(s) has(-ve) not been recovered.

Supporting Statistics

In addition to the plotting functionality of RecruitPlotEasy, a collection of basic statistics are generated upon the creation of a plot. The statistics RecruitPlotEasy produces are ANIr, breadth of coverage, and Truncated Average Depth (TAD).

ANIr is the average nucleotide identity of reads to the reference sequence at or above a particular percent identity. ANIr is weighted by the length of alignment or the entire length of reads, depending on the user’s choice.

Breadth of coverage is the percent of positions within a genome that are covered at least one time by reads mapping at or above a particular percent identity. RecruitPlotEasy reports breadth of coverage as a cumulative measure, meaning that any loci covered by a read mapping

at 100% identity are also considered covered at 99% and below in addition to whatever loci are newly covered by each decreasing percent identity bin.

TAD is measure of average depth of coverage that excludes loci with both extremely low and extremely high depths of coverage. By default, TAD is calculated over the middle 80% of depths (TAD-80). That is, the depth of coverage for each locus in the genome is found including non-covered positions as zeros, the depths are sorted in ascending order, and both the bottom and top 10% of depth values are excluded before a mean is taken from the remainder. For example, if a genome had 100 bases, the 10 loci with the highest depths of coverage and the 10 loci with the lowest depths of coverage would be removed, and the average of the middle 80 positions would be taken. RecruitPlotEasy allows the user to select the percentiles to exclude from top and bottom, meaning that while TAD-80 is the default metric, a TAD-90 value, excluding the top and bottom 5% of depths instead of the top and bottom 10%, is supported alongside any other range. As with breadth of coverage, TAD is reported cumulatively across the descending range of percent identity bins covered in a plot.

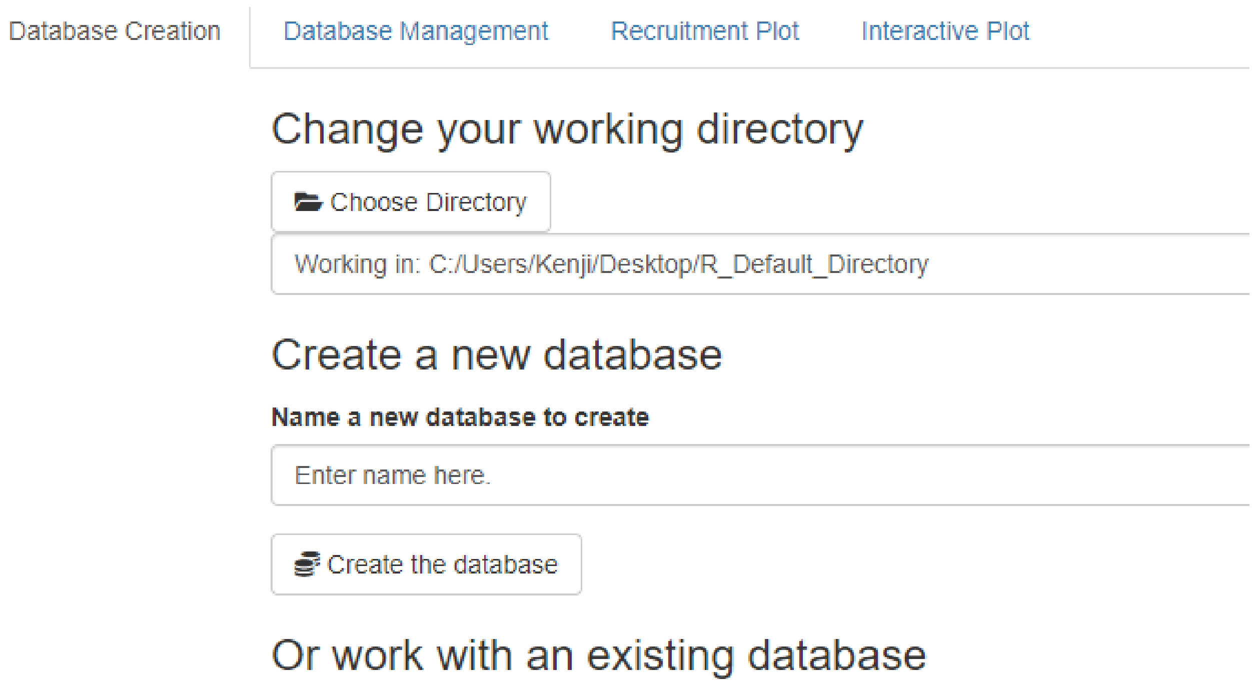

Supplemental figure 8: **The 4 tabs used by RecruitPlotEasy's GUI to divide the program's workflow into simple steps for the user, shown atop a truncated view of the database creation tab.** By default, the database creation tab is selected upon launching the GUI. The creation of a database and the addition of reads, genomes or MAGs, and genes to new or existing databases is done on the database creation page. The database management page provides information on a selected database and allows a user to give RecruitPlotEasy instructions on how to select reads for all plots. The recruitment plot and interactive plot tabs allow for the creation of

recruitment plots for genomes in a selected database as still images and as interactive, annotated plots, respectively.

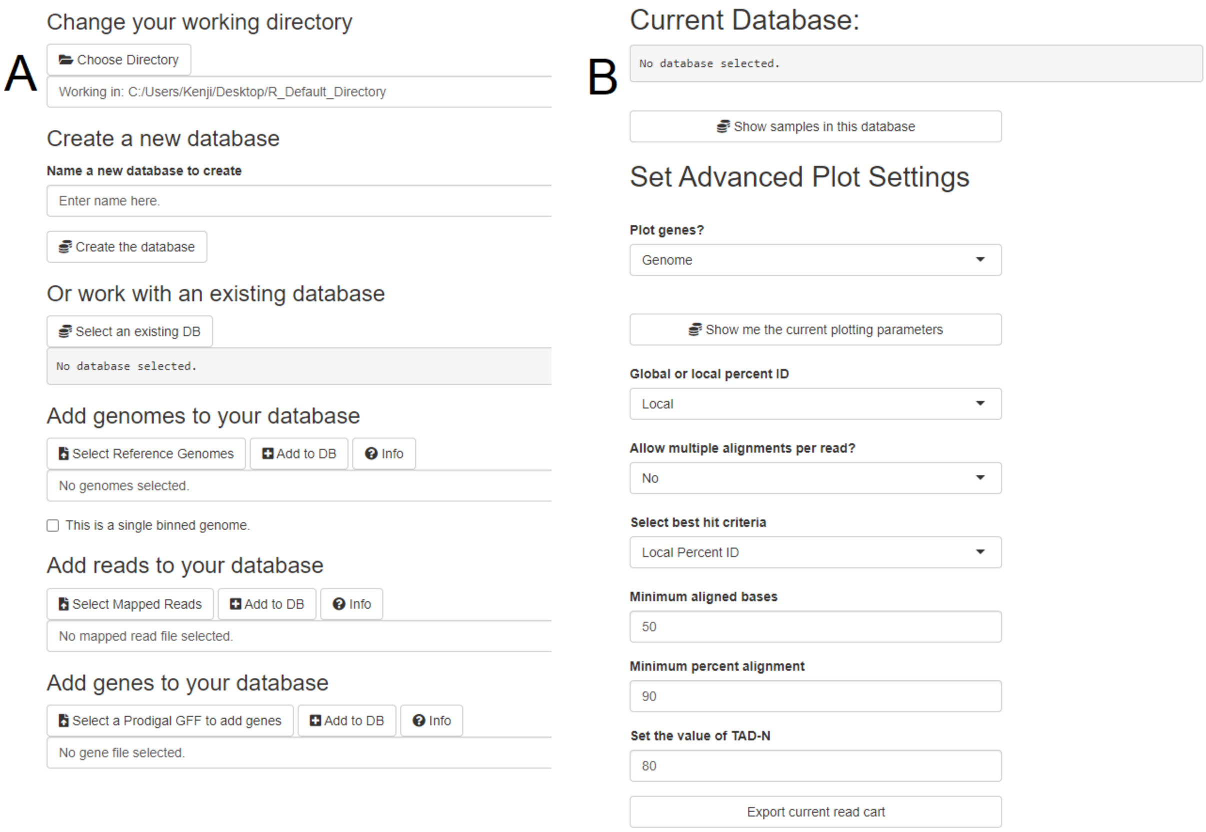

Supplemental figure 9: **The options available on (A) the database creation tab and (B) the database management tab.** Both tabs also have a text report on actions taken, which are not shown. In the database creation tab, a user may select a directory in which to save databases, plots, and data, name and create a database, select an existing database to reuse or add more data to, and add genomes or MAGs, reads in SAM, BAM, or tabular BLAST formats, and add genes in GFF format as produced by Prodigal. The database management tab allows a user to inspect their database, to make plots show genes if any are available to a given genome, to alter advanced read filtering options, to change the percentile range over which TAD is calculated, and to export the current read cart to filtered files in the same format as inputs. The advanced read filtering options control whether RecruitPlotEasy will allow a read to align more than once and by what criteria a best match will be selected if they are not, minimum alignment length and percent of the read which is aligned to be considered, and whether to calculate percent identity to the reference with respect to the aligned region of a read (local aligned fraction) or the entire length of the read (global aligned fraction), including unaligned regions.

Supplemental References

Pybam python library. https://github.com/luidale/pybam

Bushnell, B., J. Rood, and E. Singer. 2017. BBMerge – Accurate paired shotgun read merging via overlap. PLoS ONE. 12(10): e0185056.

Langmead, B., and S. Salzberg S. Fast gapped-read alignment with Bowtie 2. Nature Methods. 2012, 9:357-359.
